# Use of autologous tooth-derived graft material in the post-extraction dental socket. Pilot study

**DOI:** 10.4317/medoral.22536

**Published:** 2018-12-24

**Authors:** Alejandra del Canto-Díaz, Joaquín de Elío-Oliveros, Mariano del Canto-Díaz, Miguel-Angel Alobera-Gracia, Mariano del Canto-Pingarrón, Jose-María Martínez-González

**Affiliations:** 1 DDS, MSc. Master’s Degree in Dental Science, Complutense University of Madrid, Spain; 2 DDS, PhD. Professor of the Master’s Degree in Oral Surgery, Implantology and Periodontics, University of León, Spain; 3 DDS. Private Practice in del Canto Clinic, Madrid, Spain; 4 MD, DDS, PhD. Director of the Master’s Degree in Oral Surgery, Implantology and Periodontics, University of León, Spain; 5 MD, DDS, PhD. Department of Bucco-Facial Medicine and Surgery, Complutense University of Madrid, Spain

## Abstract

**Background:**

The objectives of the present pilot study are to compare via CBCT the alveolar contraction suffered both vertically and horizontally between the control group and the group using autologous dental material (ADM), as well as to study the densitometric differences between both post-extraction sockets.

**Material and Methods:**

A split-mouth study was performed in n = 9 patients who required two extraction of single-rooted teeth deemed suitable for deferred rehabilitation with osseointegrated implants. Two groups were formed — a control group, in which the post-extraction socket was not filled, and an ADM group, in which the alveolar defect was filled with freshly processed autogenous dental material. Both dimensional and densitometric analyses of the alveoli were performed in both groups immediately after surgery (baseline), as well as 8 weeks and 16 weeks later.

**Results:**

The mean height of alveolar bone loss was: VL (Control 1.77 mm, loss of 16.87% of initial alveolar height; ADM 0.42 mm, loss of 4.2% of initial alveolar height), HL-BCB (Control 2.22 mm, ADM 0.16 mm, *p*= 0.067 at 16 weeks). The mean bone loss of the vestibular width (VL-BCB) was much higher in the control group (1.91 mm at 1 mm, 1.3 mm at 3 mm, and 0.89 mm at 5 mm) than in the ADM group (0.46 mm at 1 mm, 0.21 mm at 3 mm, 0.01 at 5 mm, *p*=0.098 at 16 weeks). At 16 weeks, densitometric analysis of the coronal alveolar area revealed a bone density of 564.35 ± 288.73 HU in the control group and 922.68 ± 250.82 HU in the ADM group (*p*=0.045 ).

**Conclusions:**

In light of these preliminary results, autologous dentine may be considered a promising material for use in socket preservation techniques.

** Key words:**Ridge preservation, dimensional height and width changes, post-extraction socket, tooth extraction, autogenous particulate dentine graft.

## Introduction

When a tooth is lost, lack of stimulation of the residual bone results in a decrease in the trabeculae and bone density in this area, along with loss of width in the buccal bone and a subsequent loss of height in the volume of the alveolar process. These risks are particularly significant throughout the first 8 weeks (1).

Bone height decreases progressively by 25% during the first year after tooth loss, with a total of 4 mm of height lost during this first year post-extraction. The changes in vascularisation as a result of bone resorption are also important, with intrabony vascularisation shifting to centripetal periosteal vascularisation (2-5).

These dimensional changes that occur in the alveolar process may make it difficult to place an implant in a proper three-dimensional position (6).

Given the presence of soft tissue and the buccal bone wall, Elian *et al.* (7) created a system for classifying the tooth to be extracted in order to evaluate whether alveolar preservation is necessary or if immediate placement of the implant would be possible after extraction.

Alveolar preservation techniques seek to reduce the amount of bone volume lost after a tooth extraction in order to achieve enough bone volume to enable both aesthetic and functional prosthetic reconstruction after implant placement.

Grafting materials can act through three different mechanisms that promote healing of the post-extraction socket: osteogenesis, osteoinduction and osteoconduction (8).

All studied biomaterials have shown the ability to reduce the bone resorption suffered in the buccal alveolar cortical bone after extraction. Resorbable biomaterials (DFDBA, FDBA, DBM, B-TCP, calcium sulphate, etc.) necessitate implant placement within a period of time no longer than 6 months, as bone resorption is partly conditioned by functional demands. Non-resorbable biomaterials have been shown to produce excellent osseointegration and improve the long-term stability of bone crest volume (9).

Advances in tissue engineering and stem cell science have led to the development of new techniques for bone regeneration in the maxillofacial area.

Dentine has been an important topic of study due to its potential use as a bone substitute, as it has a mineral content higher than any material derived from bone. Moreover, dentine is similar to autogenous bone in two respects: it is both osseo-compatible and osteoconductive, thereby providing a physical matrix for the deposition of new bone. For the aforementioned reasons, dentine is considered an ideal bioactive material for hard tissue regeneration (10,11).

It is well established in the literature that proteins with a similar weight to bone morphogenetic proteins (BMPs) are abundant in tooth substance. BMPs help promote the differentiation of mesenchyme cells into odontoblasts and ameloblasts.

These proteins can improve the osteoinductive properties of bone substitutes if they can be successfully retained during the processing of the material studied in this experiment.

The objectives of the present pilot study are to use CBCT to compare the alveolar contractions suffered both vertically and horizontally between the control group and the study group using autologous dental material (ADM), as well as to study the densitometric differences between both post-extraction sockets.

## Material and Methods

-Human resources 

Subjects of the present study were individuals who were candidates for treatment and rehabilitation with dental implant therapy and who were patients at the Dental Practice of the Master’s Degree in Oral Surgery, Implantology and Periodontics of the University of León in Spain.

In this split-mouth clinical pilot study, the studied cases were selected and treated under the supervision of a single clinician between February 2016 and February 2017.

The study was carried out in accordance with the principles of the Declaration of Helsinki regarding research on human beings. All individuals received a thorough explanation of the study and signed their written informed consent before being included in the sample.

All individuals received a careful medical assessment as a preliminary evaluation before the surgical procedure, including a review of their dental records. Radiographic and tomographic images were taken of all patients.

Inclusion criteria.

•Healthy adult patients (over 18 years of age).  

•Patients who required extraction of two single-rooted teeth for periodontal reasons (teeth with an impossible prognosis), root caries or fractures who were also candidates for posterior replacement with an osseointegrated implant.  

•Integrity of the alveolar walls after tooth extraction.  

•Patients who accepted the study conditions, signed their written informed consent, and agreed to attend the scheduled follow-up appointments.  

Exclusion criteria.

•Patients with endocrine/metabolic disorders that might affect bone regeneration.  

•Patients with acute or chronic processes, whether general or local.  

•Patients with pathologies that might be exacerbated by the surgery itself, or by subsequent intraoperative and/or postoperative medication.  

•Patients who had taken bisphosphonates.  

•Patients who smoked over 10 cigarettes per day.  

•Lack of one or more alveolar walls after tooth extraction.  

-Study design 

A split-mouth clinical trial was carried out in a total of 9 patients who required dental extractions in single-rooted teeth for periodontal reasons or other dental reasons, and who were also eligible for rehabilitation with osseointegrated implants on a deferred basis. Two groups were formed.

•Control group, in whom the alveolar bone defect was not filled.

•ADM group, in whom the alveolar bone defect was filled with freshly processed autologous dental material (ADM).

In both groups, the bone socket was sealed using a collagen membrane.

-Surgical approach 

Tooth extraction: The extractions were performed non-traumatically using manual syndesmotomes or via attachment to a piezosurgery in order to avoid alveolar ridge alterations at the time of the extraction. A thorough alveolar curettage was subsequently carried out.

In those cases in which the extracted tooth had a root canal treatment, this was not used as donor material in the ADM group for the purposes of alveolar preservation.

Preparation and processing of the dental material: Once the dental material had been obtained and used as an alveolar filling material in selected cases, the preparation and processing of the material was carried out as indicated below.

**Figure 1:**
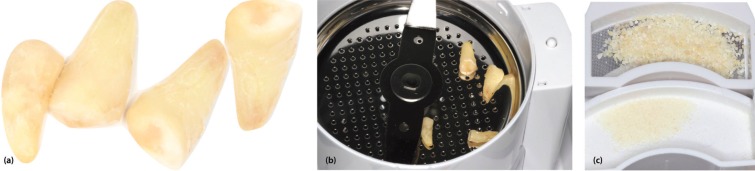
(a) Dental fragments after removal of decayed or discoloured dentine, periodontal ligament and dental plaque. (b) Fragments in the Smart Dentin Grinder® chamber. (c) Particles of dentine material of between 300–1200 microns.

1. Using fissure or flame tungsten carbide burs: removal of crowns or fillings of any kind (composites resins or amalgams), decay, or discoloured dentine, periodontal ligament and/or dental plaque (Fig. 1).

2. Rinsing with a sterile physiological saline solution and subsequent drying with an air syringe.

3. Crushing of dentine fragments in a crushing chamber capable of grinding the roots into particles of 300 and 1200 microns. The particles were ground for 3 separated using vibration for 20 seconds. Any particles smaller than 300 microns were discarded (Fig. 1).

4. The particulate material obtained was soaked for 10 minutes in a sterilised glass container with 0.5 molar of NaOH with 20% ethanol. This process results in the dissolution of organic remains, bacteria and toxins found in dentine.

5. Washing in a sterile saline solution clogged with phosphate.

**Figure 2:**
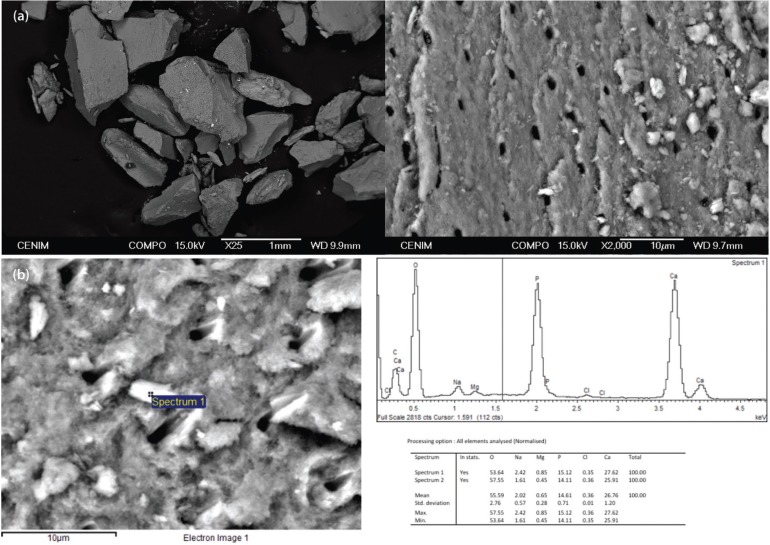
Images of the obtained bone substitute taken under an optical microscope. The prismatic and irregular appearance of the dentine fragments can be seen here, and at a higher magnification the dentinal tubuli can be identified. (b) Electron microscope image in which the atomic composition of the dentine sample can be observed; in other words, the ratio of calcium to phosphate is similar to that of bone.

Dentine samples were analysed on a preliminary basis using an optical microscope and an electron microscope (Fig. 2).

Alveolar preservation: Once curettage of the post-extraction socket had been carried out, the following steps were followed in accordance with the group in question.

•Patients in the control group did not have the alveolar bone defect filled.  

•In the ADM group, the alveolar bone defect was filled with freshly processed autogenous dentine material.  

In both groups, the socket was sealed with a 15 x 20–mm collagen membrane and the edges sutured using a 5/0 monofilament suture.  

-Dimensional analysis of the dental socket 

**Figure 3:**
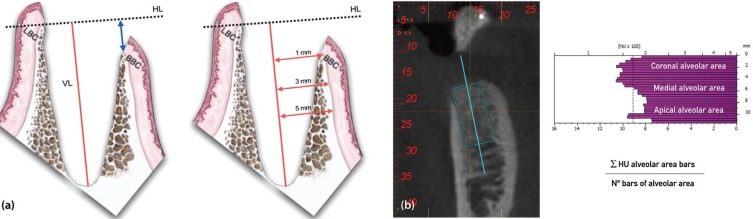
(a) Vertical and horizontal socket measurements. Vertical measurements: HL (horizontal line), VL (vertical line; tooth’s axis.), BCB (buccal cortical bone), LCB (lingual cortical bone). Horizontal measurements: HL (horizontal line), BCB (buccal cortical bone), LCB (lingual cortical bone). (b) BTI Scan software figures showing bone density in Hounsfield units (HU) and the formula used to calculate such HU units in the coronal, medial and apical alveolar areas.

Through CBCT imaging, measurements of the following dimensional parameters were taken immediately post-surgery, at 8 weeks and at 16 weeks (Fig. 3).

•Vertical measurements.

•VL distance: From the bottom of the socket to the most crestal area of the lingual cortical bone.  

•HL–BCB distance: Height difference between LCB (lingual cortical bone) and BCB (buccal cortical bone).  

•Horizontal measurements:  

•Distance from VL to BCB at 1 mm (crestal level).

•Distance from VL to BCB at 3 mm (crestal level).

•Distance from VL to BCB at 5 mm (crestal level).  

-Densitometric analysis of the dental socket 

The bone density of the regenerated bone in the post-extraction socket was determined through calculation of the Hounsfield units in the coronal, medial and apical alveolar area at 8 weeks and 16 weeks post-surgery.

The densities of each of the alveolar areas (coronal, medial and apical) were obtained using the provided formula (Fig. 3). The total number of existing bars in the figure was divided into three in order to obtain the number of bars for each alveolar area (the formula denominator).

The numerator corresponds to the summation of the HU of each bar belonging to its corresponding area. The total was divided by the number of bars in the alveolar area.

In this way, the average HU of each of the alveolar areas (coronal, medial and apical) was obtained for both the control and study sockets at 8 and 16 weeks after surgery.

## Results

-Nine individuals were selected for the study sample.

Throughout the study process, three patients were excluded (cases 4, 5 and 7), as they decided not to wait the amount of time needed for their implant rehabilitation. Therefore, the remaining total sample size was 6, three women and three men (n = 6).

-Age and gender

The mean age of the sample was 47.6 years with a standard deviation (SD) of ± 9.04, a minimum age of 39 years and a maximum of 62 years. With regard to gender, half of the sample subjects were women (3) and the other half were men (3).

-Dimensional analysis of the dental socket

**Figure 4:**
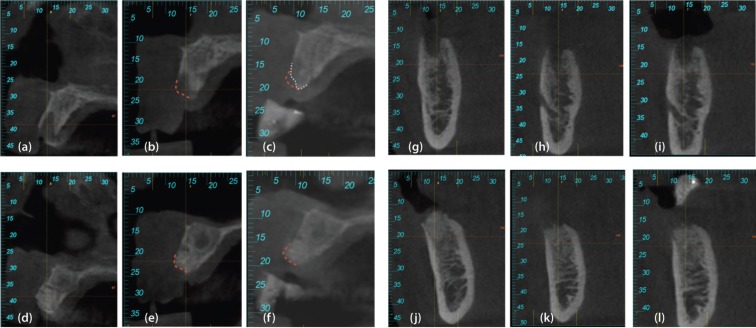
Case 1, left. (a) CBCT section of the baseline control socket (immediately post-surgery). (b) CBCT section of the control socket at 8 weeks. (c) CBCT section of the control socket at 16 weeks. (d) CBCT section of the baseline ADM socket (immediately post surgery). (e) CBCT section of the ADM socket at 8 weeks. (f) CBCT section of the ADM socket at 16 weeks. Case 2, right. (g) CBCT section of the baseline control socket (immediately post surgery). (h) CBCT section of the control socket at 8 weeks. (i) CBCT section of the control socket at 16 weeks. (j) CBCT section of the baseline ADM socket (immediately post-surgery). (k) CBCT section of the ADM socket at 8 weeks. (l) CBCT section of the ADM socket at 16 weeks.

The results, expressed in means, as well as an overall assessment, can be found in  Table 1 and  Table 2. Figure 4 shows some CBCT images.

**Table 1:**
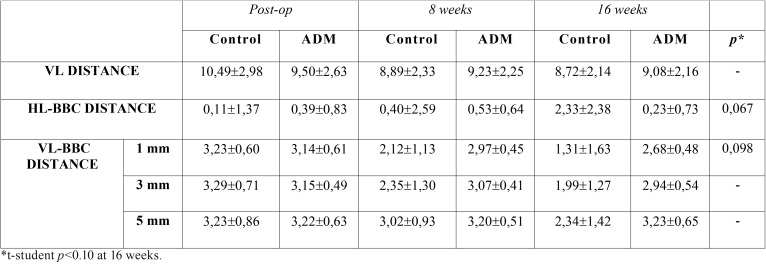
Mean values and standard deviation of the dimensional analysis of control and study (ADM) sockets immediately following surgery, at 8 weeks and at 16 weeks.

**Table 2:**
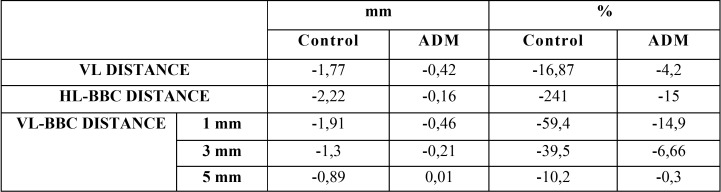
Overall assessment of the CBCT dimensional study 16 weeks later. The minus sign (-) signifies loss of height or width.

VL distance: The control group lost 1.77 mm, while the ADM group lost 0.42 mm.

HL-BCB distance: A decrease of 2.22 mm in the buccal cortical area was observed in the control group; the ADM group saw a resorption of 0.16 mm. (*p*=0.067 at 16 weeks)

VL-BCB distance at 1, 3 and 5 mm: The loss of buccal width was higher in the control sockets than in the ADM sockets, with these findings being particularly significant at 1 and 3 mm (*p*=0.098 at 16 weeks).

In the control group, a loss of 1.91 mm at 1 mm and 1.3 mm at 3 mm was observed. At 5 mm, a loss of 0.89 mm was recorded.

In the ADM group, at 1 mm a loss of 0.46 was measured, with 0.21 mm at 3 mm and 0.01 mm at 5 mm.

-Densitometric analysis of the dental socket

 Table 3. shows the mean densities.

**Table 3:**
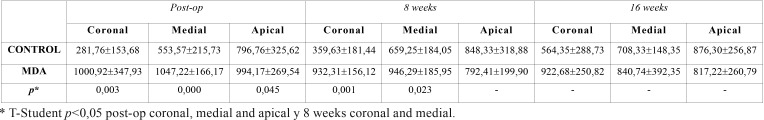
Mean values expressed in Hounsfield units (HU) of the control and ADM sockets immediately after surgery (baseline values), at 8 weeks and at 16 weeks.

Coronal alveolar area: Immediately following surgery, the density obtained in the control group was 281.76 HU with a SD of ± 153.68; in the ADM group, the density was 1000.92 HU with a SD of ± 347.93 (*p*=0.003).

At 8 weeks, in the control group the recorded density was 359.63 HU with a SD of ± 181.44, and in the ADM group the density was 932.31 HU with a SD of ± 156.12 (*p*=0.000).

At 16 weeks, the control group showed a density of 564.35 HU with a SD of ± 288.73, and the ADM group 922.68 HU with a SD of ± 250.82 (*p*=0.045).

Medial alveolar area: Immediately following surgery, the density obtained in the control group was 553.57 HU with a SD of ± 215.73, and in the ADM group the density recorded was 1047.22 HU with a SD of ± 166.17 (*p*=0.001).

At 8 weeks, the control group obtained a density of 659.25 HU with a SD of ± 184.05, and the ADM group 946.29 HU with a SD of ± 185.95 (*p*=0.023).

At 16 weeks, the density obtained in the control group was 708.33 HU with a SD of ± 148.35, and the ADM group showed a density of 840.74 HU with a SD of ± 392.35 *p*<0.05 immediately surgery, and at 8 weeks).

Apical alveolar area: Immediately following surgery, the density obtained in the control group was 796.76 HU with a SD of ± 325.62, and the ADM group showed a density of 994.17 HU with a SD of ± 269.54.

At 8 weeks, the control group obtained a density of 848.33 HU with a SD of ± 318.88, and the ADM group 792.41 HU with a SD of ± 199.90.

At 16 weeks, the control group obtained a density of 876.30 HU with a SD of ± 256.87, and the ADM group 817.22 HU with a SD of ± 260.79.

## Discussion

There are studies currently underway aimed at evaluating the effectiveness of different bone grafting materials, in which the histology of the socket and the dimensional changes that occur when using different types of materials are both analysed after treatment.

However, scientific evidence does not provide clear guidelines with regard to the most adequate biomaterial for these purposes.

The main bone substitutes are synthetic mineral materials and allogeneic and xenogeneic bone. Nevertheless, autogenous bone is still considered the gold standard, as it is the only substitute that shows osteoinductive, osteoconductive and osteogenic properties, despite the fact that it must be harvested and the possible morbidity that this entails.

Lindhe (12) asserts that the inorganic and organic composition of dentine is very similar to bone. In particular, its organic matrix is also dominated by Type I collagen fibres and presents non-collagen proteins such as phosphoproteins, osteocalcin, proteoglycans and glycoproteins. Consequently, both past and recent experimental studies have focused on the use of dentine as a potential bone substitute in some defect models. Essentially, it has been demonstrated that dentine, when used in particles or blocks, showed more osteoconductive and osteoinductive properties, and it became involved in the bone remodelling process (13-15).

The present split-mouth clinical trial was carried out to evaluate the use of autologous graft material of dental origin in alveolar preservation techniques, using CBCT imaging to compare the alveolar contractions suffered horizontally and vertically between the control group and the autologous dental material (ADM) group,, in addition to comparing the densitometric characteristics of post-extraction sockets in both groups.

Two possible applications for the use of dentine graft as a bone substitute have been proposed. One is its use as a graft requiring processing via demineralisation procedures 15 similar to those used in the manipulation of allogeneic bone. The other is as a fresh autologous material, in which the dentine is used without prior demineralisation. This second alternative is the one described in this study. On the other hand, some authors are using it in particle (15) or block (17-19) form.

Kim *et al.* (15) introduced the extracted tooth as a new bone graft material to outweigh the disadvantages of allografts, xenografts and synthetic grafts. Their dentine graft preparation system includes a demineralisation process. Even though the demineralised dentine exposes the growth resulting from the matrix and differentiating factors for healthy osteogenesis, newly formed bone and demineralised residual dentine are weak when it comes to supporting implant anchorage. Conversely, the SDG (Smart Dentin Grinder®) procedure enables dentine’s preparation into bacteria-free particles using freshly extracted autologous teeth, which are ready for immediate use as biomaterials.

The results of the dimensional analysis in this study reveal that the control group lost 1.77 mm on average (16.87% of the socket’s initial height), coinciding with most of the studies published by Ten *et al.* (19) and Hämmerle *et al.* (20), in which the vertical loss is over 1.5 mm. Similarly, the information in this study coincides with the literature in that most of this loss occured during the first 8 weeks. In the present study, 83% of loss occurred during the first 8 weeks. However, in the ADM group, only 0.41 mm of bone loss, or 4.2% of the initial socket height, was observed,.

On the other hand, with regard to the buccal cortical height, a mean decrease of 1.44 mm was observed in the control sockets, while the ADM sockets maintained their cortical height. The buccal width loss was higher in the control sockets than in the ADM sockets, notably at 1 and 3 mm, in which losses of 1.91 mm (control) at 1 mm and 1.3 mm (control) at 3 mm were observed. The ADM group saw an average bone loss of only 0.47 mm at 1 mm and 0.21 mm at 3 mm.

Likewise, Joshi *et al.* (21) obtained similar dimensional results, with reduction of crest height being significantly lower at 0.28 ± 0.13 mm (compared to 0.41 mm in the present study) in the dentine autologous graft as opposed to other areas with no graft, which saw a reduction of 2.60 ± 0.88 mm (*p* < 0.05), (compared to 1.77 mm in the present study). With regard to changes in width, these were statistically significantly higher in the non-grafted sites, at 2.29 ± 0.40 mm (1.3 mm in the present study), than in the sites with autologous dental graft, at 0.15 ± 0.08 mm (*p* < 0.05) (0.21 mm in the present study). It is worth noting that the study carried out by the aforementioned authors does not specify the methodology used for obtaining these measurements.

On the other hand, Schwarz *et al.* (17,18) carried out studies in foxes using dentine root blocks instead of particles. These authors observed that the grafts of premolar roots were replaced more homogeneously by newly formed bone than in the autogenous bone grafts, concluding that the roots of extracted teeth revealed a structural and biological potential to work as an autograft alternative to autogenous bone. In their study conducted in foxes, Becker *et al.* (19) also compared dentine root blocks with autologous bone, and after implant placement they concluded that both grafts supported the early stages of osseointegration.

With regard to the limitations of the present study, it should be noted that it is purely experimental, and additional studies are therefore required in order to corroborate these findings using a higher sample size.

Moreover, additional research is needed to identify the bone remodelling properties of dentine through histological and histomorphometric study, as well as a research to compare dental autogenous material with the most used biomaterials in bone regeneration.

## Conclusions

- The dimensional contraction of the post-extraction socket in the ADM group was lower than the control group at 16 weeks after surgery, both vertically and horizontally.

- The densitometric values (HU) in the ADM group remained stable and homogeneous between the three areas, being equal to the control group after 16 weeks, with the exception of the coronal area, which continued to show higher values.

- As observed in the preliminary results of this study, autologous dentine is a promising material for use in socket preservation techniques.
